# How audiences express emotions towards influencer short videos: Sentiment analysis of ‘my second uncle’

**DOI:** 10.1371/journal.pone.0341797

**Published:** 2026-01-27

**Authors:** Rongchun Xiao, Xiaoyu Guo, Xueying Xie

**Affiliations:** 1 School of Humanities and International Communication, Ningbo University of Technology, Ningbo, China; 2 School of Accounting, Nanjing Audit University, Nanjing, China; Universidad Santiago de Cali, COLOMBIA

## Abstract

As short videos dominate digital communication, this study examines audience emotional expression in this evolving media landscape. Focusing on the popular Chinese video “My Second Uncle”, we collected bullet subtitles—user comments displayed on-screen—and related comments. Using Latent Dirichlet Allocation (LDA), we identified key themes, while SnowNLP and ChatGPT facilitated an in-depth emotional analysis. Findings show a nuanced audience “gaze” toward the protagonist, with a strong trend of embracing his inspirational story, which generated predominantly positive emotional responses. Unlike traditional “gaze” research, engagement here was active and voluntary, with viewers using innovative online language. The study also reveals that influencer-driven short videos can quickly foster positive group sentiments, underscoring their potential for emotional healing and mental well-being.

## Introduction

In the rapidly evolving digital media landscape, internet celebrities play a pivotal role in shaping online culture and user engagement, particularly on short-video platforms. By June 2022, China’s short-video user base had surged to 962 million, underscoring the medium’s extensive reach and influence [1]. Internet celebrities have become central to this phenomenon, with their content profoundly shaping Chinese users’ digital experiences and preferences.

However, the essence of their influence lies not merely in broad dissemination but in their capacity to trigger deep emotional resonance and collective gaze. A prime example is the viral short video “Three Days Back in the Village, My Second Uncle Cured My Mental Exhaustion”, which spread widely across major social platforms in mid-2022 and evoked intense emotional responses. Such moments of viral transmission demonstrate that influencer videos have become focal points for public emotional expression and convergence.

Therefore, this study aims to transcend superficial observations of the viral phenomenon and delve into the emotional interaction mechanisms between its content and audience. Specifically, using the “My Second Uncle” video as a case study, it explores how audiences emotionally engage with viral short videos, focusing on expressions of empathy and spectator behaviors. By analyzing audience sentiment data on Bilibili, this research not only aims to reveal how viewers establish emotional connections with content and how these emotions shape online interactions but also seeks to examine the evolving dynamics of ’gaze’ and empathy within the new media context of short videos. While existing research has examined emotional expressions within videos, deeper analysis is needed on the dynamic processes of audience sentiment over time and across topics, as well as their implications for public discourse. The findings offer insights for media practitioners, policymakers, and mental health professionals seeking to understand the nuanced emotional narratives of digital audiences.

This paper proceeds as follows: Section 2 provides a literature review, Section 3 outlines the methodology, Sections 4 and 5 present the results of LDA and sentiment analysis, and Section 6 discusses “gaze” and empathy in short videos. Section 7 concludes the study.

## Literature review

### Research on public emotional expression and analysis in the network

With the rise of social media and short-video platforms, online emotional expression has become more dynamic and complex. Research primarily examines the features and factors influencing emotional expression on the internet, revealing the diversity and interactivity of audience emotions in short-video contexts. On user-generated content (UGC) platforms, real-time engagement through bullet subtitles, posts, and replies allows audiences to express emotions through symbols, numbers, emojis, and graphics [2], forming an “emotional atmosphere” with rapid dissemination and collective resonance [3]. This emotional resonance, often quick to ferment public opinion, strengthens community sentiment and cohesion around specific events [4]. Emotional expression, therefore, functions as an exploration of an event’s meaning, creating a collective response from the “bottom up” [3,5,6].

The social context of each platform also shapes emotional expression: on closed platforms like WeChat, expression tends to be conservative, while on open platforms like Weibo, it is more direct and intense [2]. Additionally, emotional diffusion varies by emotion type, with anger spreading more rapidly than others [7]. Economic factors also play a role, with higher economic development correlating with reduced positive emotional expressions and mitigation of fear [8]. Emotional expression, while beneficial for consensus-building, can also amplify extreme emotions, posing challenges for public opinion governance [9].

Building on existing studies, this paper uniquely examines the emotional expressions of audiences within the context of influencer short videos, providing a novel analytical perspective and research value. While previous literature has largely focused on the general characteristics of emotional expression on UGC platforms, including the use of symbols, emojis, and varied modes of expression in open-platform environments, this study emphasizes the unique influence and emotional interaction mechanisms specific to influencer content creators. Influencer short videos, which are often more closely aligned with the everyday lives of ordinary people, tend to generate deeper emotional resonance and stronger emotional transmission chains—a specificity that has not yet been widely addressed in research.

### Theoretical perspective and reflection of gaze

Gaze theory builds upon the Western concept of “visual centrism” and explores “seeing” from diverse perspectives. It examines how people form deep, often unconscious, connections with others through the act of gazing, positioning oneself in the viewpoint of the other, and projecting feelings such as self-regard, fear, or desire onto them [10]. Hegel was among the first to discuss gaze in Phenomenology of Spirit, considering how human beings initially construct a subject-object relationship through mutual “seeing” [11].

Sartre, Lacan, and Foucault each contributed foundational insights into gaze theory from the standpoint of vision. Sartre argued that the self and others confirm their existence through gaze, emphasizing, from an existentialist view, that meaning is established in the act of seeing, which, in turn, affirms the essential role of visual experience in human subjectivity [12]. Lacan, from a psychoanalytic perspective, proposed a nuanced relationship between the gaze and the human eye, where the eyes signify the subject and the gaze represents the object. He saw the gaze as a projection of the subject’s desires, a phenomenon that can lead the viewer to experience lust and fixation during the act of gazing [13,14]. Foucault further developed the theory by linking power with gaze, creating a framework of vision-knowledge-power, exemplified by his concept of the “medical gaze” [15].

Starobinski posited that gaze functions as a social behavior and an organizing force in social relationships, whereby the innate human impulse to see and desire to explore opens a “second vision.” This expanded vision reveals a field of infinite potential,metaphorically similar to lifting a veil, which Starobinski argues can lead to a power dynamic akin to the right of interrogation or control [16]. Vision, subjectivity, and power are therefore central to gaze theory. Danny also viewed gaze as a visual behavior rooted in social power dynamics, integral to understanding “social identity” [17].

At the same time, some scholars have critiqued the traditional understanding of gaze by seeking to dismantle its inherent power dynamics. Their work focuses on examining gaze as it relates to women and racial identities. Hooks suggested that “seeing” challenges established authority and can be a form of confrontation [18]. Gurnham (2018) has extended this critique by encouraging subjects to engage in a reflective, oppositional gaze, reshaping the viewer-object dynamic. This opposition reveals the inter play of rights, desires, and resistance that exists between the viewer and the viewed, framing a visual relationship that touches upon issues of gender, race, and culture and contributes to a broader theoretical approach in postmodern cultural studies [19].

In the digital era, Xu argues that gaze theory has evolved with the rise of live streaming, blurring traditional gender boundaries in the dynamics of looking and being looked at. Now, men, too, are increasingly subjects of the gaze [20]. This shift reflects a broader transformation in the logic of visibility under digital capitalism. As Schröter [21] proposes, the proliferation of visual content and algorithmic circulation has given rise to what he terms the “digital capitalist gaze,” in which subjects are constituted through their visibility in media networks. Chang [22] further deepens this analysis by showing how motion capture technologies render the human body as dynamic data, destabilizing traditional boundaries between the observer and the observed, and evoking a sense of algorithmic uncanny. Liang [23] introduces the notion of “co-gazing” to describe how the traditional disciplinary surveillance of the “Panopticon” is reconfigured in digital environments: individuals no longer simply resist being seen, but increasingly internalize and even pursue visibility as a form of social value. This framework resonates with broader discussions on affective labor and self-surveillance on digital platforms. These changes also complicate the role of empathy in mediated environments. Liu [24] points out that digital technology is not inherently an enabler of empathetic communication, but rather the structural condition in which such communication occurs. While digital affordances such as comment systems and emotional tagging may shape the expression and reception of emotion, their actual impact on empathy remains ambiguous.

### Research on empathy

Empathy is a fundamental ability inherent in human nature [25]. It is distinct from sympathy, which is often a form of pity lacking objectivity. In contrast, empathy involves a rational emotional response—experiencing and understanding what an other person thinks and feels [25]. Raboteg-Saric identified two types of empathy: vicarious emotional responses to others and the self-perception of others’ inner states. The first, known as “Emotional Empathy,” refers to an empathetic emotional response [26].

Empathy is not a singular emotion but a complex psychological process. Neuroscientific research has shown that empathy is grounded in physiological mechanisms. Rizzolatti and colleagues discovered the existence of “mirror neurons” in the human nervous system, which play a crucial role in empathetic responses [27]. These neurons allow individuals to immediately feel the emotions conveyed by others when confronted with intense emotional stimuli. This neural mechanism helps form emotional connections between individuals, enabling them to synchronize their emotional responses [28]. The neural network involved in empathy matures with age [29].

However, some researchers have raised concerns about the role of mirror imitation in empathy. While mirror imitation may help humans understand others’ intentions, they argue that simply imitating actions does not necessarily imply a full understanding of those behaviors or a mental alignment with the emotions of others. If mirror imitation is central to empathy, how do mirror neurons determine which behaviors to “imitate” to facilitate empathetic connections [30]. In response, Wu [28] proposed that “love”underpins empathy, with its expression varying according to the objects of empathy and the situations in which it arises. He also emphasized that communication is key to cultivating empathy, which in turn plays a critical role in fostering a global community with a shared future.

While much existing research has focused on the “panopticon” framework, which typically views audiences as passive objects of surveillance, new trends in short videos featuring internet celebrities suggest a shift in the dynamics of “gaze.” In these videos, audiences are no longer just passive observers but actively express emotions and engage in self-disclosure. This creates a “super-panopticon,” where traditional subject-object roles are blurred. This research explores two key questions: (1) How do audiences “gaze”at figures like the “second uncle” in influencer videos? (2) How do audiences generate empathy through comments and bullet subtitles? These inquiries aim to shed light on the nature of audience “gaze” and emotional expression—an area that has been largely unexplored in existing literature.

## Methodology

### Framework

**Fig 1:**
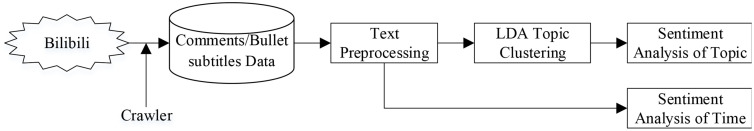
Research framework.

The research framework of this paper, as illustrated in Fig 1, begins with the collection of comments or bullet screen data from the Bilibili platform. The collected text undergoes preprocessing: segmentation using the jieba library, removal of stop words, and elimination of meaningless symbols. Subsequently, the processed text is subjected to topic clustering using the Latent Dirichlet Allocation model [31] for generating topic probability distributions. LDA, based on the bag-of-words model, probabilistically selects topics and words within topics. By clustering words, LDA produces two distributions: topic-word probability distribution and comment-topic probability distribution. For the comment-topic probability distribution, the sentiment value of each comment is computed using the SnowNLP, thereby calculating the percentage of positive and negative sentiments under each topic. Comments are sorted by time, and SnowNLP is utilized to study changes in sentiment over time. Bullet screen data is sorted by progress bar, investigating changes in sentiment along the progress bar.

### Experimental data and preprocessing

**Fig 2:**
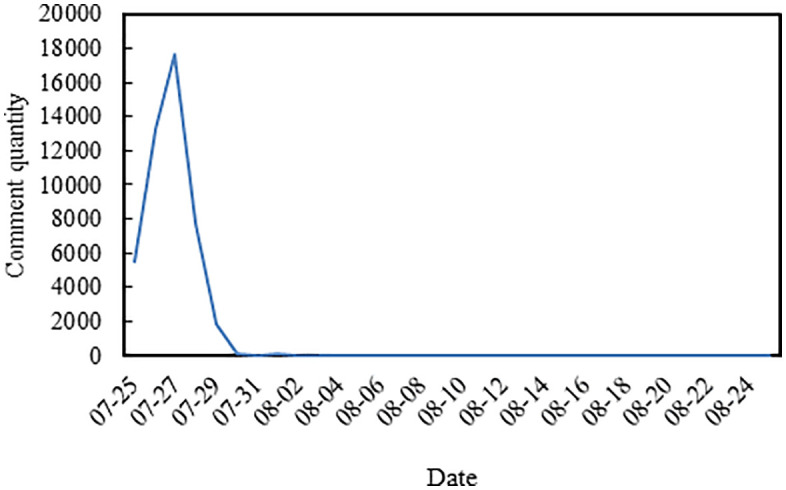
Trend of comment quantity fluctuation over time.

The study gathered primary comments and bullet subtitles from “My Second Uncle” on Bilibili, spanning from July 25 to August 25, 2022. A Python crawler program was developed for this purpose, resulting in the collection of 46,436 comments and 3,000 bullet subtitles. The data used in this study were collected from publicly available content on the Bilibili platform. All personally identifiable information was removed during data processing. Fig 2 illustrates the fluctuation in the number of comments overtime, showing a sustained low level after the 29th. The significant peak in comments observed around July 27–28 corresponds to the period when the “My Second Uncle”video achieved widespread virality on Bilibili. This surge in engagement was likely fueled by its powerful narrative resonating deeply with public sentiment, combined with active platform recommendations. The sharp decline in comments after July 29th and the subsequent sustained low levels are characteristic of the life cycle of viral content.This trajectory is generally attributable to several factors: the natural dissipation of the initial emotional impact, the shifting of public attention towards broader societal discussions or new media controversies, and the dynamics of platform algorithms, which often reduce the visibility of peak-performing content to promote the exposure of newer uploads.

**Fig 3:**
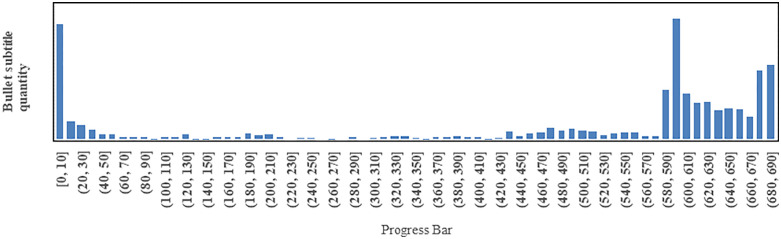
The variation in bullet subtitle quantity as it relates to the progress bar.

Fig 3 displays the variation of bullet subtitle quantity along with the progress bar,grouped in intervals of 10 seconds.

Before the experiment commenced, data preprocessing was conducted. The comment texts often contain novel emoticons, which are rich in emotional polarity. Bilibili’s back end stores emoticons in text format. To prevent the emoticons from being segmented into multiple words with different emotional directions, all emoticon words from the comments were added to the segmentation dictionary. Additionally, besides removing stop words, some meaningless special symbols were eliminated. Because the“@” symbol is typically followed by a Bilibili user’s ID, which contributes little to sentiment recognition tasks, regular expressions were used to remove the “@” symbol and its subsequent ID names. Using the Gensim library in Python, an LDA model was constructed. Preprocessed words were fed into the LDA model for training, and the model’s effectiveness was evaluated using coherence metrics [32].

LDA topic clustering analysis of comments and bullet subtitles in ‘my second uncle’

## Topic clustering of comment data

### LDA clustering of comment data from the first five days

**Fig 4:**
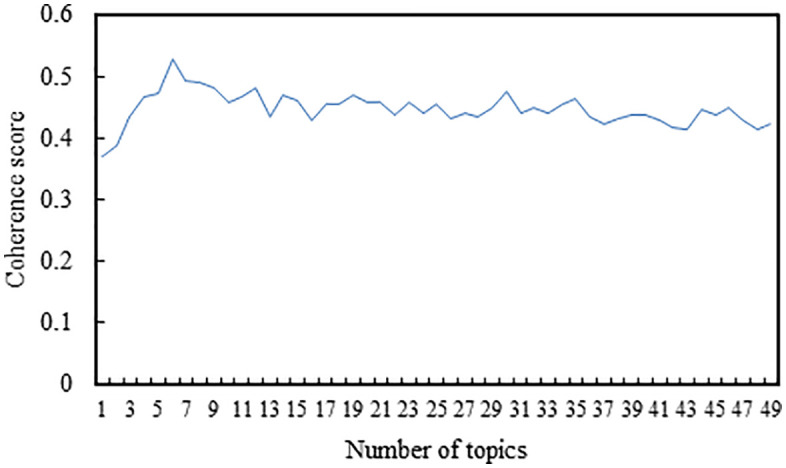
The variation in coherence scores relative to the number of topics in the comment data from the first five days.

**Fig 5:**
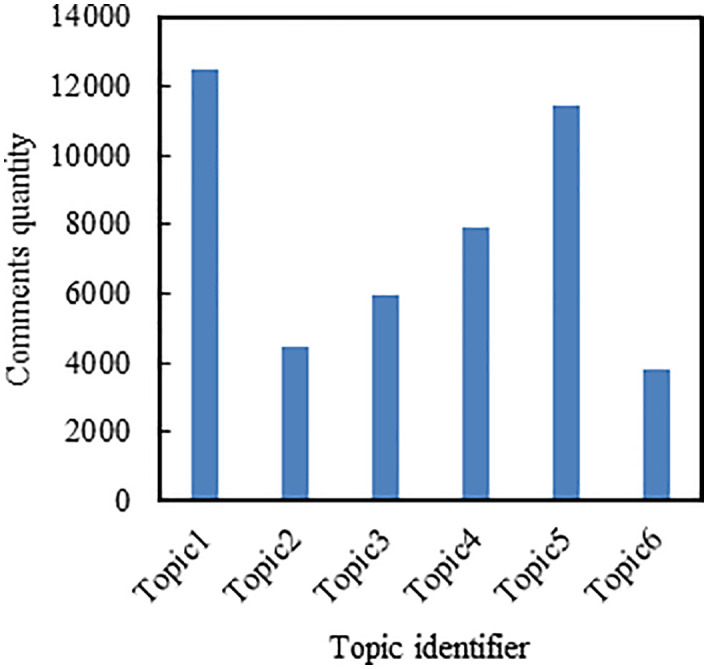
The distribution of comment quantities across different topics based on the first five days of comment data.

The LDA model was implemented using the Gensim library. We adopted the default hyperparameters provided by Gensim, where the Dirichlet priors were set to α = 1.0/num topics and β = 1.0/num topics. Using the comment data from the first five days for LDA topic clustering, Fig 4 illustrates the variation of coherence scores with the number of topics. From the graph, it is evident that when the number of topics is set to 6, the LDA model achieves the highest coherence score. Subsequently, as the number of topics continues to increase, the change in coherence score becomes minimal and does not surpass the maximum coherence value. Therefore, the optimal number of topics is determined to be 6. Under the scenario of 6 topics, the topic-word probability distributions are generated, providing the vocabulary distribution for each topic. Table 1 displays the topic-word distributions for the first five days of data, wherein the words under each topic are sorted in descending order of probability. The top 10 most relevant words for each topic are selected and used to label the topics. Retrieve the topic number for each comment data and tally the comment count for each of the six topics, as depicted in Fig 5.

**Table 1:** Topic-word distribution of comment data from the first five days.

Topic identifier	Topic label	Word items
Topic1	Fate	suffer, uncle, banality, person, life, great, live, justice
Topic2	Emotion	mental exhaustion, cry, heal, uncle, three days, pure, play, great, smile, true
Topic3	Gesture	cry, video,..., time, doge, check, feel, finish, like, again
Topic4	Talents	uncle, respect, do, again, now, think, person, feel, remember, little
Topic5	Experience	uncle, video, true, article, support, story, great, person, moved, want
Topic6	Vicissitudes	person, pity, happy, life, past, memory, others, first, likes

### LDA clustering of comment data from the first month

**Fig 6:**
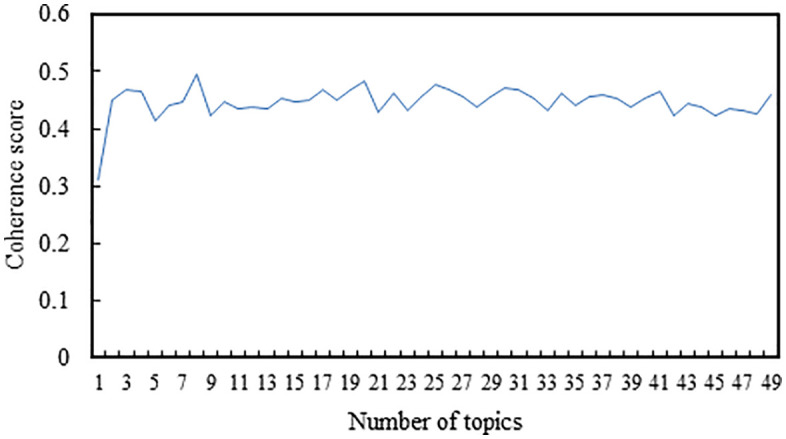
The variation in coherence scores with the number of topics in the comment data over the first month.

Using the first month of comment data for LDA topic clustering, Fig 6 displays the variation of coherence scores with the number of topics. From the graph, it is evident that when the number of topics is set to 8, the LDA model achieves the highest coherence score. Subsequently, as the number of topics increases, the change incoherence is minimal and does not surpass the maximum coherence value. Therefore,the optimal number of topics is determined to be 8. Under the scenario of 8 topics, the topic-word probability distributions are generated, providing the vocabulary distribution for each topic. Table II presents the topic-word distributions for all comments, wherein the words under each topic are sorted in descending order of probability. The top 10most relevant words for each topic are selected and used to label the topics. By comparing and analyzing Tables 1 and 2, it can be observed that with the passage of time, the number of topics that the audience is interested in also increases.

**Table 2:** Topic-word distribution of comment data from the first month.

Topic identifier	Topic label	Word items
Topic1	Gesture	Crying, too, so good, touching, really, awesome, second uncle, copy writing, shocking, likes
Topic2	Introspection	, grandpa, salute, remember, do, also, see, now, teacher, home
Topic3	Memory	Person, regret, happiness, life, live, past, look back, other, world, need
Topic4	Emotion	..., fair, great, video, play, smile, laugh and cry, ten thousand, three in a row
Topic5	Motivation	Cow, alive, victory, film, doge, fight, against all odds, ok, make up your mind, poison
Topic6	Experience	Second uncle, people, life, think, feel, video, life, really, hope, know
Topic7	Talents	Video, copy writing, respect to second uncle, second uncle, up, coin, up, life, like, method
Topic8	Fate	Ordinary, great, cry, support, call, second uncle, video, life, real, feeling

**Fig 7:**
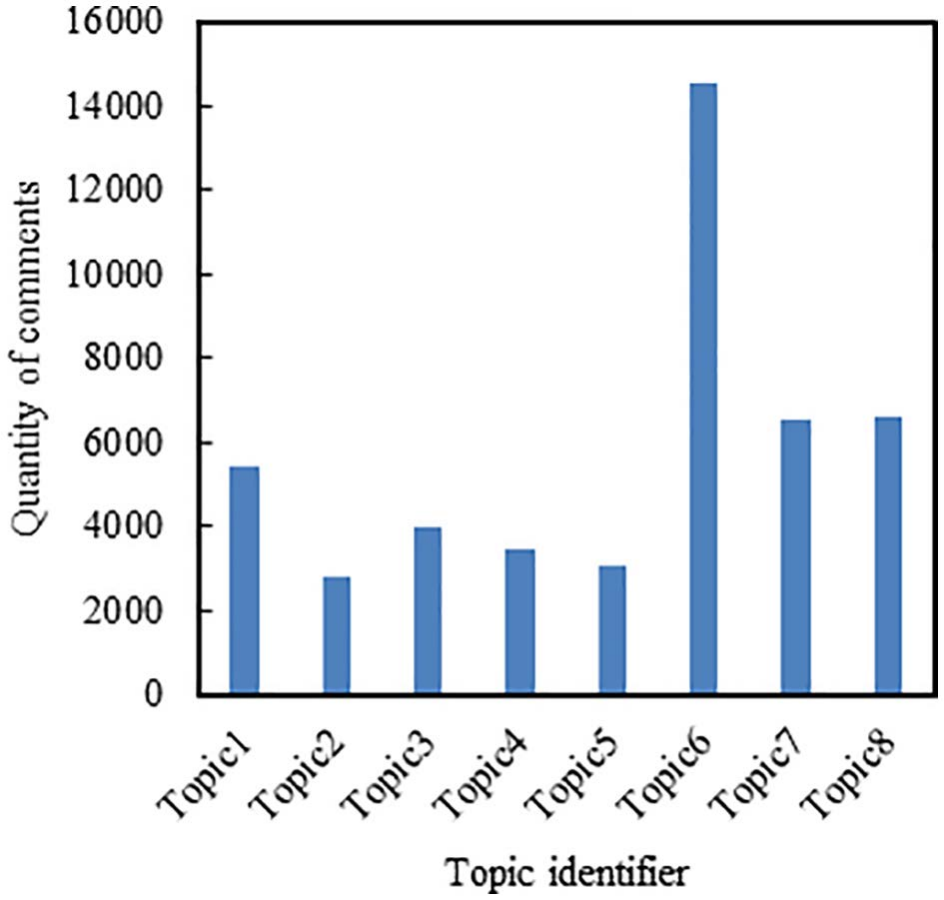
The distribution of comment quantities across different topics based on the first month’s comment data.

Retrieve the topic to which each comment belongs and obtain the count of comments for each of the eight topics, as shown in Fig 7.

### Topic clustering of bullet subtitles

**Fig 8:**
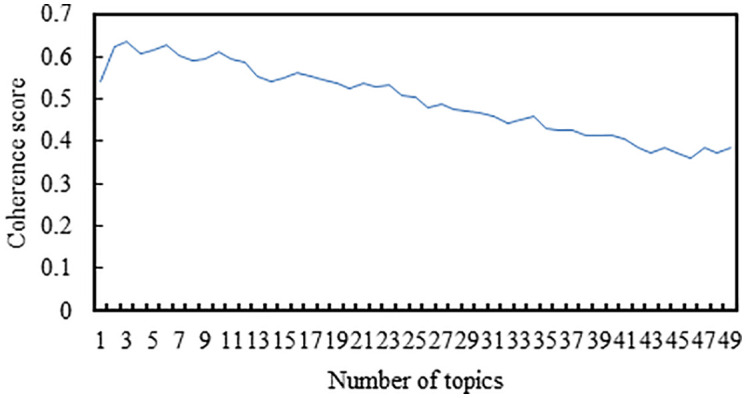
The variation in coherence scores with the number of topics in the bullet subtitle data.

LDA is applied in the topic clustering of bullet subtitles data. Fig 8 shows how the coherence varies with the number of topics. The coherence of the LDA model is the largest when the number of topics is set to 3. The coherence gradually decreases as the number of topics continues to increase. Hence the optimal number of topics is 3. The topic-term probability distribution is generated when the number of topics is 3. Then the distributed vocabularies of each topic are obtained.

Table 3 shows the topic-term distribution of the data. The words of each topic are sorted from large probability to small one. The top 10 most relevant words for each topic are selected and the topics are labeled concerning them.

**Table 3:** Topic-word distribution of the bullet subtitles data.

Topic identifier	Topic label	Word item
Topic1	Gesture	Respect to the second uncle, awesome,...,!, coins more than likes, Waizi, village, colonel, Mr.
Topic2	Fate	To, hah, cry, ordinary, people, suffering, too, great, powerful, video
Topic3	Talents	The second uncle, really, people, life, now, way, always, salute, warrior, almighty

**Fig 9:**
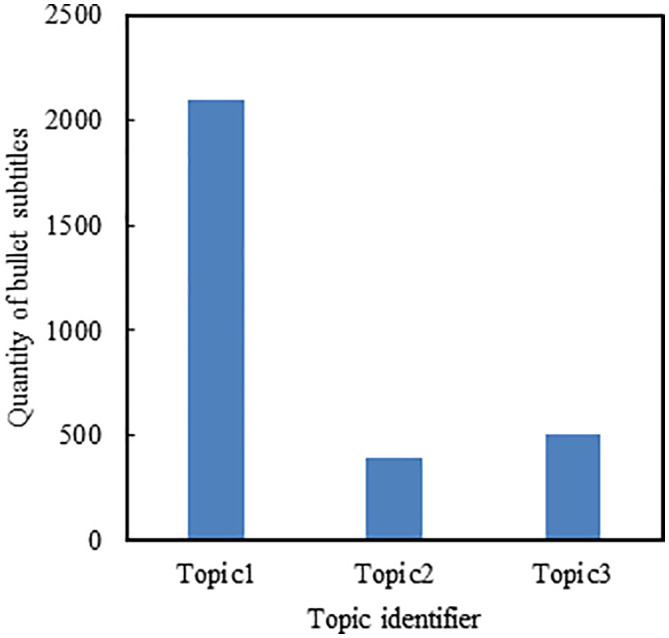
The number of bullet subtitles across different topics.

Each bullet subtitle is classified into the corresponding topic. Fig 9 shows the bullet subtitle number of the three topics.

## Sentiment interpretation

### Sentiment analysis of comments

SnowNLP was utilized to compute the sentiment value of the comments using its built-in sentiment analysis module, which is trained on user-generated content.Comments surpassing 0.5 were classified as positive, while those below 0.5 were considered negative. We also adopted a more nuanced approach to sentiment analysis by incorporating a broader spectrum of complex emotions. As shown in Table 4, we designed prompt instructions for ChatGPT to classify user comments into ten fine-grained emotion categories: nostalgia, sadness, praise, like, anger, surprise, fear, motivation, neutral, and reflection.

**Table 4:** Prompt design.

Prompt type	Prompt content
Role assignment	Assume you are an expert in analyzing emotional information in Chinese video comments.
Task description & requirements	Please identify the emotional category of each comment on the video *“Back to the Village for Three Days, My Second Uncle Cured My Spiritual Exhaustion.”* Requirements: (1) The candidate emotion categories include: *nostalgia, sadness, praise, like, anger, surprise, fear, motivation, neutral,* and *reflection*. Definitions of each category are shown in Table 5. Choose the label with the highest estimated probability as the final classification; (2) Output the results in a spreadsheet, assigning one label per comment.
Refusal strategy	If a comment cannot be judged, please indicate so explicitly.

**Table 5:** Definitions of emotion categories.

Label	Description
nostalgia	Memory-evoking contexts, such as childhood, countryside, or returning home.
sadness	Expressions of sorrow, heartbreak, emotional tears, and empathetic pain.
praise	Clear positive recognition of people or actions, such as admiration, respect, or resilience.
like	Expressions of support or fondness for the video content or creator (e.g., “liked it,” “watched again,” “gave a coin”).
anger	Clear expressions of anger, injustice, or condemnation.
surprise	Expressions of astonishment or unexpected reactions (e.g., “didn’t expect that,” “so shocking”).
fear	Expressions of fear, anxiety, or psychological discomfort.
motivation	Inspired feelings to take action or work harder, such as “I want to try harder” or “this helped me get back up.”
neutral	Comments with no obvious emotional tone, mere factual statements, or lacking sufficient information.
reflection	Philosophical or contemplative thoughts, such as “life is unpredictable” or “this is the reality of the world.”

### Sentiment analysis on the thematic dimension

Calculating the sentiment values of a total of 46,048 comments from July 25th to July29th, we obtained 37,754 positive comments and 8,294 negative comments, accounting for 81.99% and 18.01% respectively. Thus, the proportion of comments with positive sentiment significantly outweighs those with negative sentiment. Table 6 presents the distribution of emotional categories in user comments over five days. While neutral comments are the most common, the emotion of motivation stands out among the meaningful categories, indicating that many viewers felt inspired or encouraged by the video.

**Table 6:** Emotion distribution over the first five days.

Date	Nostalgia	Sadness	Praise	Like	Anger	Surprise	Fear	Motivation	Neutral	Reflection
7/25	1041	262	161	69	4	58	63	322	3317	199
7/26	3060	563	333	144	5	130	117	903	7497	541
7/27	4634	727	368	153	13	152	143	1283	9475	744
7/28	2275	220	133	60	8	40	70	478	4188	279
7/29	569	30	26	10	2	12	19	72	1030	46
Sum	11579	1802	1021	436	32	392	412	3058	25507	1809

**Fig 10:**
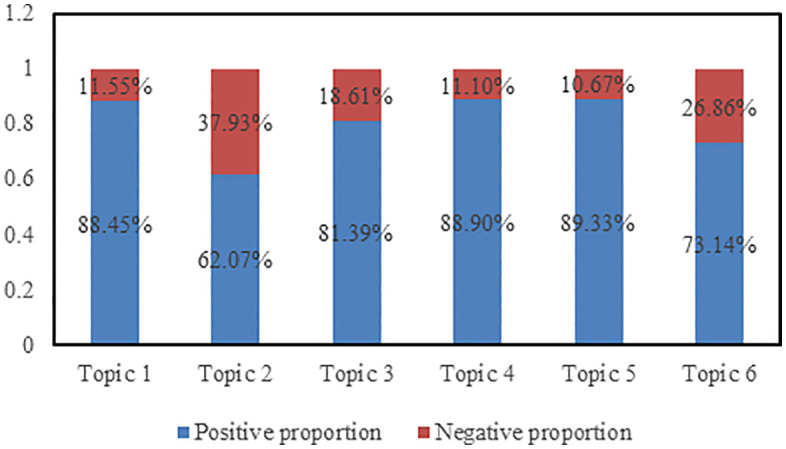
The positive and negative percentages for the six topics of comments from the past five days.

Clustering the comments from the first five days resulted in six topics, with the percentage of positive and negative sentiment for each topic shown in Fig 10. It can be observed from the figure that the proportion of positive sentiment comments exceeds that of negative sentiment comments for all topics. The highest proportion of positive sentiment comments is for Topic 5, while the lowest is for Topic 2. This indicates that audiences express predominantly positive sentiment in their comments; however, compared to other topics, there is a higher prevalence of negative sentiment projected onto Topic 2.

### Sentiment analysis on the temporal dimension

**Fig 11:**
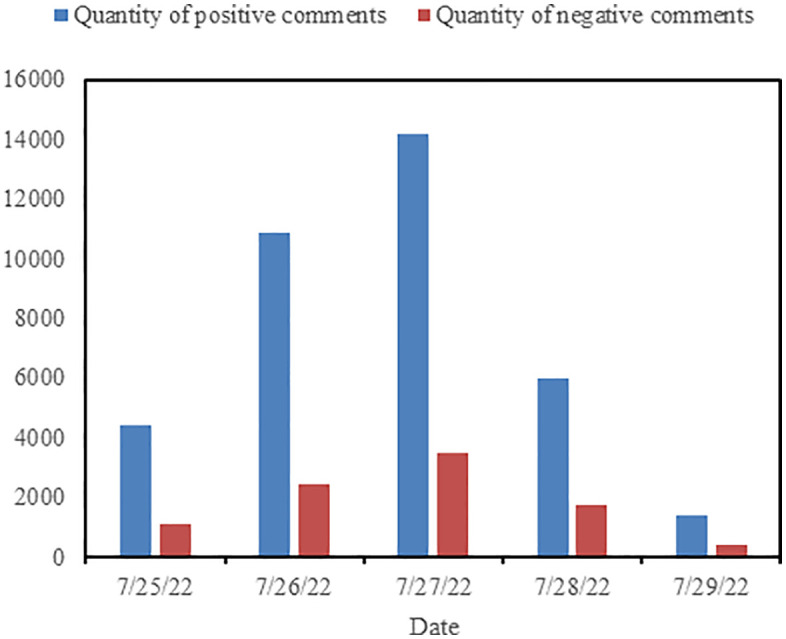
The number of positive and negative comments over the first five days.

**Fig 12:**
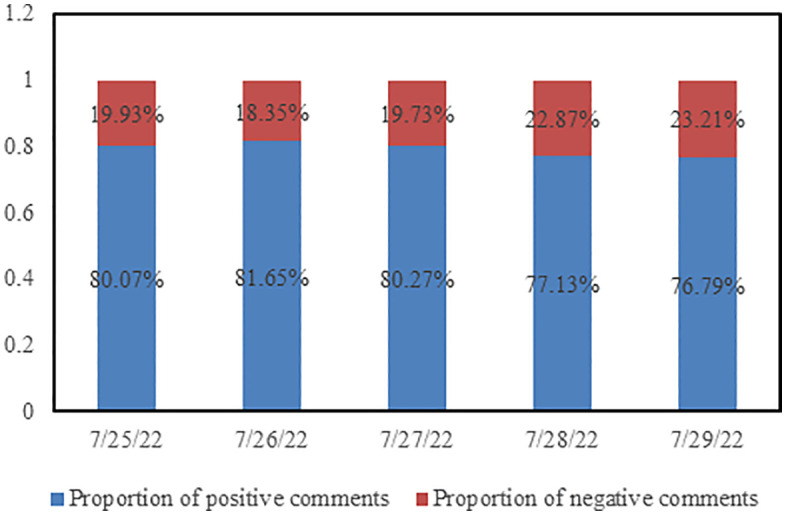
The proportion of positive and negative sentiments in comments from the first five days.

To illustrate the temporal evolution of sentiment in comments, a bar chart was plotted with dates on the horizontal axis and the number of comments on the vertical axis, as shown in Fig 11. From the graph, it can be observed that both the number of positive sentiment comments and negative sentiment comments exhibit an increasing followed by decreasing trend over time, although the number of negative sentiment comments each day is significantly lower than that of positive sentiment comments. Fig 12 displays the percentage of positive and negative sentiment comments over the five-day period. It can be seen from the graph that the proportion of positive and negative comments remains relatively stable for the first three days, while the proportion of negative comments gradually increases on July 28th and 29th.

### Sentiment analysis of bullet subtitles

For the bullet subtitles, we set the sentiment classification threshold of the SnowNLP to 0.44 to distinguish between positive and negative sentiment orientations. This threshold was determined based on an analysis of representative samples. Specifically, manual examination revealed that a sentiment score of 0.440219 corresponded to a clearly positive textual expression, whereas a score of 0.4390476 reflected an evidently negative sentiment. Given the minimal difference between these two values and their consistent semantic polarity, a threshold of 0.44 was selected as a conservative cutoff point to ensure robust classification performance.

### Emotion analysis based on topics

**Fig 13:**
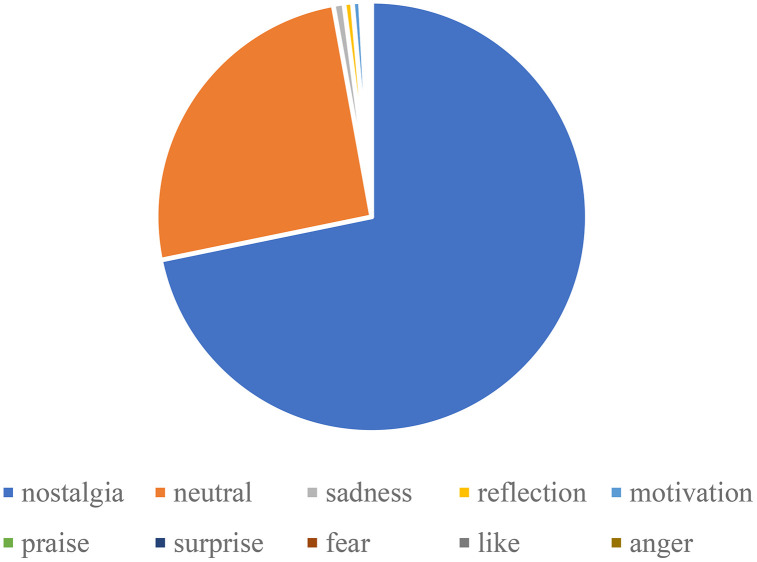
Distribution of emotional categories in bullet comments.

Emotional analysis of bullet subtitles reveals 2,751 positive and 249 negative comments, accounting for 91.70% and 8.30% respectively. Therefore, the proportion of positive bullet subtitles far exceeds that of negative bullet subtitles. The complex emotions distribution is shown in Fig 13. We can see that the majority of bullet comments are categorized as nostalgia, reflecting a strong collective memory and emotional resonance triggered by the video. In contrast, other emotions such as sadness, motivation, and reflection appear much less frequently.

**Fig 14:**
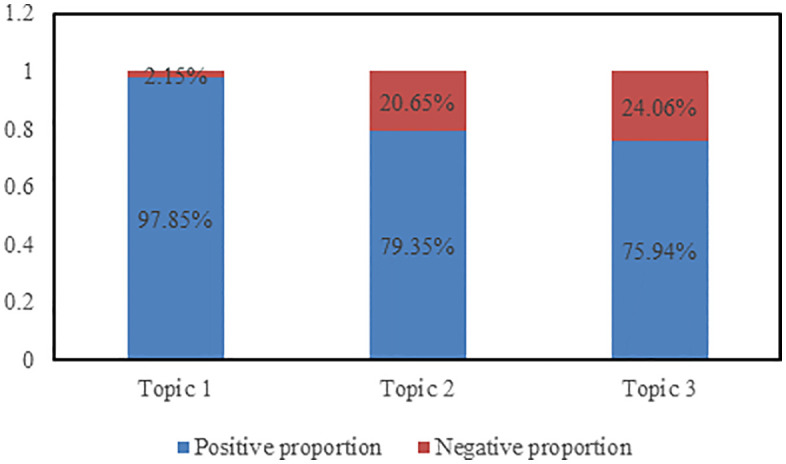
The proportion of positive and negative sentiments across the three topics of bullet subtitles.

By clustering bullet subtitles, three topics have been identified, with the proportion positive and negative sentiment for each theme shown in the following figure. As depicted in Fig 14, the proportion of positive bullet subtitles exceeds that of negative bullet subtitles for all topics. The highest proportion of positive bullet subtitles is observed in Topic 1, while the lowest is in Topic 3. This suggests that audiences predominantly express positive emotions in bullet subtitles; furthermore, compared to other topics, audiences project more positive emotions towards Topic 1.

### Emotion analysis based on the progress bar of the video

**Fig 15:**
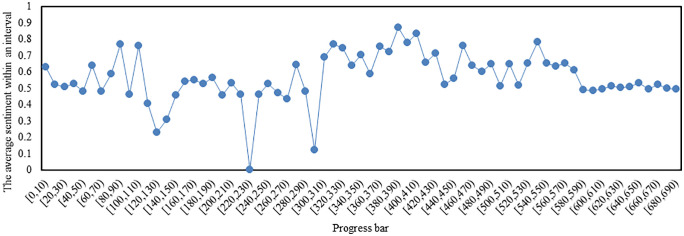
The average sentiment of bullet subtitles varies with the progress bar (Note: In the interval [220, 230), no bullet subtitles are present, resulting in a sentiment value of 0).

**Fig 16:**
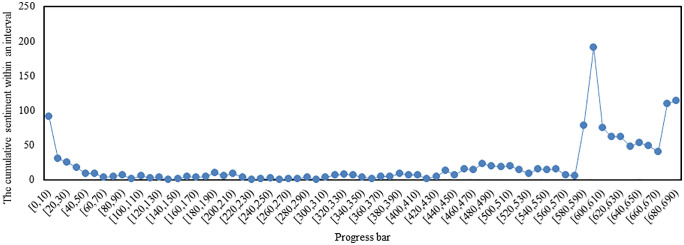
The cumulative sentiment of bullet subtitles varies along the progress bar.

At intervals of every 10 seconds, calculate the average and cumulative values of emotion within each 10-second period, and plot the trend graphs as shown in Figs 15 and 16,respectively. From Fig 15, it can be observed that bullet subtitles mostly fluctuate within the positive sentiment range, with only a small number of time intervals exhibiting negative bullet subtitles, among which the sentiment of bullet subtitles in the[290,300) interval is the most negative, while the sentiment of bullet subtitles in the[380,390) interval is the most positive. The [290,300) interval and preceding scenes(barrages have a certain lag) describe the emotional life of the protagonist’s uncle,involving financial issues. The [380,390) interval and preceding scenes describe the protagonist’s grandmother’s life, involving perspectives on life and death (‘Illness and aging are necessary performances between life and death’), as well as the uncle’s attitude and actions towards ‘elderly care’.

From Fig 16, it can be observed that the cumulative sentiment remains stable within the interval [10, 580), reaching its peak in the interval [590, 600). As seen in Fig 3 earlier, the interval [590, 600) has the highest number of bullet subtitles, which contradicts our intuitive understanding. Through observation, it is evident that many users watch the video multiple times and leave more bullet subtitles at the beginning and end of the video. Therefore, the number of bullet subtitles is generally highest at the beginning and end of a video. Looking at the interval [590,600) and the preceding content, we can see: “He is such a genius, just born in the wrong era. He should have been nurtured by the country, and his fame should have spread far and wide. It’s such a pity, too regrettable.” However, my second uncle is not regretful, “The second happiest person is the one who never looks back.”

### “Gaze” practice and empathetic communication in internet celebrity short videos

The practice and pattern of audience “gazed” at the second uncle

The research found that there is a certain degree of bias in the audience’s gaze towards the short video “My second uncle”. This bias is mainly reflected in the greater attention given to abnormal emotional life, traditional elderly care, and my second uncle’s optimistic attitude towards life. Audiences also tend to express emotions, forming a specific “gaze” practice and pattern.

This paper, through an empirical study of the short video “My second uncle,”combined with thematic classification and sentiment analysis of comments and bullet screen messages, summarizes the patterns of audience gaze on the protagonist, my second uncle, as shown in Fig 16. Bentham used the “panopticon” to describe modern power. Poster proposed the concept of the “Super Panopticon,” suggesting that,compared to the early “panopticon,” the “Super Panopticon” is based on digital technology. Its surveillance scope is broader, and its methods are more flexible.Individuals are monitored and disciplined unconsciously, willingly accepting this surveillance. Each individual participates in a self-construction process, transforming themselves into the subjects of surveillance in the Super Panopticon. Bauman believes that surveillance in modern society is a fluid, liquid form of monitoring. The mode of discipline has shifted from “rational” obedience, rule-following, and conformity to“irrational” proactivity, risk-taking, experimentation, and self-assertion, as well as the pursuit of emotion, pleasure, and entertainment [33].

**Fig 17:**
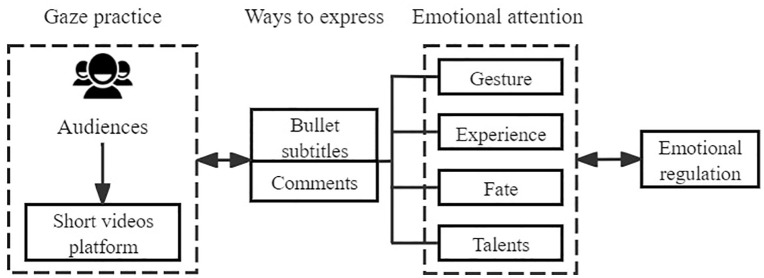
The manner in which the second uncle is ‘gazed at’ by audiences in the short video.

Irrational emotional factors are an important component of this type of fluid, liquid surveillance. Particularly in digital communication practices, highly visual and interactive gazes become a new environment for emotional expression. In light of this,the gaze patterns in the short video “My Second Uncle” can be summarized as components, communication environment, expression methods, and emotional focus, as shown in Fig 17. Meanwhile, the far right end of this mode points to emotional governance, involving the need for platforms and regulatory authorities to strengthen the management of grotesque and harmful emotional expressions in comments and bullet subtitles. Additionally, a large amount of positive emotion can serve as an important resource for empathetic communication, applicable to specific communication fields.

From the perspective of its components, the short video platform provides a liquid surveillance scenario. The audience, by watching the story of “My Second Uncle”through the short video, plays the role of the “observer”; My Second Uncle, positioned at the center of the “Super Panopticon,” assumes the role of the “observed.” Comments and bullet subtitles are the specific ways in which the audience monitors the second uncle, consciously or unconsciously projecting their own experiences, transforming them into emotional expressions.

From the perspective of the communication environment, sophisticated video editing techniques allow the audience to quickly browse through the second uncle’s life experiences, compressing time and space. The application of intelligent communication technology enables each viewer to see others’ comments and understand their emotional inclinations, creating a subjective environment for emotional expression. To a certain extent, when viewers post comments or bullet subtitles, they are influenced by both the content they are viewing and the emotional expressions of other viewers. Additionally,sentiment analysis of the comments and bullet subtitles reveals that viewers have a significantly positive emotional response to the second uncle. This positive emotion flows within and outside the video and comments, permeating the overall communicative atmosphere. It is evident that communication technology greatly constructs the environment in which viewers “gaze” at the second uncle, making him the subject of the audience’s “liquid surveillance.” Simultaneously, the analysis of viewers’ positive emotional values indicates that viewers are also being “positively disciplined” by the second uncle.

From the perspective of expression methods, the audience mainly conveys their emotions and responds to the narrative through comments and bullet subtitles. For example, in Topic 5, keywords are “bull, living, victory, movie, doge, strive, overcome all difficulties, OK, determined, poison.” Other projective expressions include terms like“hexagonal warrior” (meaning an all-rounder). Additionally, there are innovative non-verbal expressions, such as the “Protect Carrot Crying” emoji on the Bilibili platform. These methods are used by the audience to express positive emotions. This mode of expression demonstrates a willingness to accept and internalize the narrative.On one hand, the audience monitors the second uncle’s story, while on the other hand,they willingly engage in self-reflection, sharing their own experiences and feelings, thus transforming into the observed.

From the perspective of emotional focus, the audience pays attention to four types of gaze: physical symbols, life experiences, life trajectory, and talent abilities. The audience naturally sympathizes with the second uncle’s physical disability, and coupled with his exceptional talent, it creates a strong contrast that elicits intense emotional investment from the audience. The second uncle’s legendary life experiences and tumultuous life trajectory, narrated in the short video, evoke a sense of historical vicissitudes, striking a chord with the audience’s deeply buried emotional pain points,and triggering emotional resonance.

### Expanding applications of empathetic communication driven by‘gaze’ practices

Through posting and reviewing comments and bullet subtitles, the audience engages in the practice of gaze, which is a collective and emotional process. In the interaction within short videos, like-minded individuals gather to discuss, with comments and bullet subtitles reinforcing each other and flowing in a unified direction. However, this group of strangers, influenced by group thinking, may lead many to rely less on their own information and more on discussions with others or others’ viewpoints. This phenomenon, known as group polarization as described by Sunstein [34], results in a“cascade effect” that amplifies and disseminates empathetic communication. Based on the findings of this study, the second uncle is at the center of the group’s gaze in the short video. His physical symbols, life experiences, life trajectory, and talent abilities receive intense attention from the audience. Centered around the second uncle’s “inspirational story of suffering,” the audience invests positive emotions. Simultaneously,the audience completes self-gazing and mutual gazing through others’ comments and bullet subtitles. Through extensive discussion, a “cascade effect” is formed, resulting in empathy.

In the practice of “gaze” in short videos, the second uncle is the fixed object of the“gaze,” with his character image and storytelling being the focal points. Compared to traditional “gaze,” the practice of “gazing” at the second uncle reflects a relatively balanced power structure. The audience does not surveil from a superior position but engages in a mutual “gaze” as fellow ordinary people, displaying a positive motivational effect. Comments and bullet subtitles in the practice of gaze are crucial bridges guiding the audience towards empathy.

As suggested by the title of the short video “Three Days Back in the Village, My Second Uncle Cured My Mental Exhaustion,” the story of the second uncle shared through short video platforms has sparked public participation and viewership. Viewers compare and reflect on their own experiences, contemplating their mental exhaustion and seeking balance and emotional support from the second uncle’s story. In this practice of gaze, the boundaries between the “self” and the “other” are blurred because the audience often gazes at the second uncle from their own perspectives and experiences. This reflective process, which extends one’s self to others, incorporates both sympathy and empathy, thus facilitating empathetic communication. The phenomenon of short videos attracting large groups of viewers with positive emotions in a short time has become a significant resource for empathetic communication. This offers new samples and insights for related theoretical and practical research.

## Conclusion

This study analyzes audience emotional data from the Bilibili platform to reveal how viewers establish emotional connections with short video content,and how such emotions shape online interactions and to explore the dynamic evolution of ’gaze’ and empathy practices within the new media context of short videos.

Findings reveal that audiences engage asynchronously with the ordinary human stories in My Second Uncle through screens. Though not expecting responses, they persistently express emotions, forming a practice of cross-spatial emotional expression rooted in connection. Against the specific social backdrop of the COVID-19 pandemic, this story elicited collective ’gazing’. Audiences engage in self-reflection through gazing and express emotions via comments and bullet screens. This interaction sparks discussions, mutual influence, and a ‘chain reaction effect’,triggering collective emotional release and empathy within a short time frame.

Compared to previous studies on online ‘gazing’, this research demonstrates that audience gazing practices and theories in viral short videos have evolved: Audiences at the center of the ‘panopticon’ exhibit proactivity and voluntariness, actively expressing emotions through comments and bullet screens—even engaging in self-disclosure—adding new dimensions to the gazing structure. The boundaries between’surveillance’ and ‘discipline’ in gaze practices are increasingly blurred, while the rigid distinction between gaze subjects and objects gradually dissolves. New characteristics emerge collectively through social context, technology (intelligent algorithms, mobile internet), expression methods (comments and bullet screens), surveillance direction (bidirectional), and disciplinary approaches (shifting from passive to active).

Simultaneously, this study experimentally assessed the emotional value of audience comments and bullet screens, revealing the dissemination effects of influencer short videos from a viewer perspective. This provides valuable insights for understanding audience focus, expression methods, emotional trends, and optimizing platform algorithm recommendations. Findings indicate that the overwhelming majority of viewers hold positive emotions toward ‘Second Uncle’ videos and experience positive motivation, demonstrating the content’s significant positive guiding value. This suggests that evaluating short video content should not rely solely on interaction metrics but should also focus on its emotional and social significance; ‘emotional value’ can serve as an effective indicator for guiding content evaluation and platform governance.

Based on these findings, we recommend that platforms optimize algorithms to identify and promote content that offers emotional comfort and healing despite its melancholic undertones, rather than simply demoting content containing sadness or sensitive elements. Simultaneously, policymakers should establish regulatory frameworks and incentive mechanisms to encourage the production and dissemination of digitally created content that is emotionally constructive and socially positive, fostering a healthy and inclusive online media environment.

This study has the following limitations: First, this study employs a single-case analysis approach, focusing on the short video ‘My Second Uncle’ and its dissemination on the Bilibili platform during a specific period. This methodology facilitates an in-depth examination of audience interaction mechanisms within a particular cultural and platform context, with findings offering valuable reference points for similar emotionally resonant short videos. However, inherent limitations of the research method restrict the broad applicability and generalizability of the conclusions. Therefore, future research could further explore commonalities and differences in short video dissemination across platforms and sociocultural contexts through cross-cultural comparisons. Alternatively, expanding to multi-case comparisons or large-scale quantitative studies could enhance the universality and explanatory power of research conclusions.

Second, due to technical limitations, this study could not determine whether the comments and bullet subtitles were influenced by capital or platform manipulation. Future research could collaborate with multiple platforms to obtain more comprehensive user information and conduct comparative analyses of audience emotional expression data across platforms. Additionally, subsequent research could integrate effective AI-based comment detection tools to refine data screening and mitigate potential biases in the data set.

Third, this study did not address the possibility that Bilibili’s algorithm may influence the visibility of user comments. Specifically, the platform may prioritize displaying certain comments or suppressing others based on factors such as interaction metrics, posting time, or semantic content, leading to a bias in the comment data set toward specific expression types. Future research should focus on eliminating the impact of Bilibili’s algorithm on data visibility to enhance the effectiveness of sentiment analysis.

Finally, future research could also focus on gender as a significant social dynamic factor to explore its role in audience reception of such videos and emotional engagement processes, thereby deepening our understanding of audience interaction mechanisms in digital spaces.
